# What Is the Best NGS Enrichment Method for the Molecular Diagnosis of Monogenic Diabetes and Obesity?

**DOI:** 10.1371/journal.pone.0143373

**Published:** 2015-11-23

**Authors:** Julien Philippe, Mehdi Derhourhi, Emmanuelle Durand, Emmanuel Vaillant, Aurélie Dechaume, Iandry Rabearivelo, Véronique Dhennin, Martine Vaxillaire, Franck De Graeve, Olivier Sand, Philippe Froguel, Amélie Bonnefond

**Affiliations:** 1 CNRS-UMR8199, Lille Pasteur Institute, Lille, France; 2 Lille University, Lille, France; 3 European Genomic Institute for Diabetes (EGID), FR 3508, Lille, France; 4 Department of Genomics of Common Disease, School of Public Health, Imperial College London, Hammersmith Hospital, London, United Kingdom; Odense University Hospital, DENMARK

## Abstract

Molecular diagnosis of monogenic diabetes and obesity is of paramount importance for both the patient and society, as it can result in personalized medicine associated with a better life and it eventually saves health care spending. Genetic clinical laboratories are currently switching from Sanger sequencing to next-generation sequencing (NGS) approaches but choosing the optimal protocols is not easy. Here, we compared the sequencing coverage of 43 genes involved in monogenic forms of diabetes and obesity, and variant detection rates, resulting from four enrichment methods based on the sonication of DNA (Agilent SureSelect, RainDance technologies), or using enzymes for DNA fragmentation (Illumina Nextera, Agilent HaloPlex). We analyzed coding exons and untranslated regions of the 43 genes involved in monogenic diabetes and obesity. We found that none of the methods achieves yet full sequencing of the gene targets. Nonetheless, the RainDance, SureSelect and HaloPlex enrichment methods led to the best sequencing coverage of the targets; while the Nextera method resulted in the poorest sequencing coverage. Although the sequencing coverage was high, we unexpectedly found that the HaloPlex method missed 20% of variants detected by the three other methods and Nextera missed 10%. The question of which NGS technique for genetic diagnosis yields the highest diagnosis rate is frequently discussed in the literature and the response is still unclear. Here, we showed that the RainDance enrichment method as well as SureSelect, which are both based on the sonication of DNA, resulted in a good sequencing quality and variant detection, while the use of enzymes to fragment DNA (HaloPlex or Nextera) might not be the best strategy to get an accurate sequencing.

## Introduction

Diagnostic testing for monogenic forms of diabetes and obesity is of paramount importance for the patient and his/her family as it can lead to personalized medicine associated with a better life. Indeed, the patients presenting with neonatal diabetes mellitus (NDM), maturity-onset diabetes of the young (MODY), or adult onset atypical type 2 diabetes, who carry a heterozygous mutation in *ABCC8* or *KNCJ11* (encoding subunits of the ATP-dependent potassium channel at the membrane of pancreatic beta cells), can be successfully switched from an expansive and demanding insulin therapy to easy and cheap oral sulfonylureas, with much better result on glucose control [1]. Furthermore, MODY patients with a heterozygous mutation in *HNF1A* (MODY-3) can also be optimally treated with an oral sulfonylurea agent [2]. For society, it has been demonstrated that diagnosis of MODY and NDM save life and money, as degenerative complications can be avoided at lower costs [3,4].

Among the patients with severe, early-onset obesity, the carriers of a homozygous mutation in the leptin gene (*LEP*) can be treated with recombinant leptin therapy, which leads to a marked and sustained reduction in weight [5]. In countries with high levels of consanguinity, one of five severely obese patients carries a mutation in *LEP* [6]. The development of quick, reliable and cost-effective protocols for genetic diagnosis of diabetes and obesity are limiting factors for precision medicine in metabolic diseases.

Most of genetic diagnostic laboratories are currently moving from Sanger sequencing to next-generation sequencing (NGS) approaches but it is not clear which enrichment method yields better specificity and sensitivity in the field of monogenic diabetes and obesity, resulting in the most accurate variant detection.

In the present study, we aim to compare four different enrichment solutions based on the sonication of DNA (SureSelect Human All Exon, Raindance microdroplet-based PCR enrichment), or using enzymes for the fragmentation of DNA (Nextera Rapid Capture Expanded Exome, HaloPlex Custom Kit), in combination with Illumina NGS. To assess these four methods, we processed DNA samples from two MODY patients with a known causal mutation in *HNF1B*, and then we compare sequencing coverage of targeted regions and variant detection. To our knowledge, this is the first time a head-to-head technical comparison is made between these four techniques.

## Methods

### Patient Selection

We selected two patients presenting with MODY, who were previously diagnosed for a causal mutation in *HNF1B* (NM_000458.2). The study protocol was approved by local ethic committees (Cruces University Hospital—*Centro de Investigaciὀn Biomédica en Red de Diabetes y Enfermedades Metabὀlicas Asociada*, Barakaldo, Spain), and study participants signed an informed consent.

### RainDance Microdroplet-Based PCR Enrichment

A custom panel was designed to target exons and untranslated regions (UTRs; including 5’UTRs and 3’UTRs) with 50 base-pairs (bp) of intronic flanking regions, of a total of 43 genes involved in monogenic forms of diabetes (*ABCC8*, *CEL*, *EIF2AK3*, *FOXP3*, *GCK*, *GLIS3*, *HNF1A*, *HNF1B*, *HNF4A*, *INS*, *KCNJ11*, *NEUROD1*, *NEUROG3*, *PDX1*, *PTF1A*, *RFX6* and *WFS1*) and obesity (*ALMS1*, *ARL6*, *BBS1*, *BBS2*, *BBS4*, *BBS5*, *BBS7*, *BBS9*, *BBS10*, *BBS12*, *BDNF*, *CEP290*, *GNAS*, *LEP*, *LEPR*, *MC4R*, *MKKS*, *MKS1*, *NTRK2*, *PCSK1*, *POMC*, *SDCCAG8*, *SIM1*, *TRIM32*, *TTC8*, *WDPCP*), as previously described [7]. Of note, all transcripts were included in the panel. The two DNA samples were processed following the manufacturer’s protocols. Briefly, genomic DNA was sonicated (Bioruptor NGS; Diagenode, Liège, Belgium) and added to a PCR mix. Subsequently, the RainDance primer library was merged with each sample mix on the RDT1000 (RainDance Technologies, Billerica, MA, USA). The resulting emulsion was amplified by PCR and used for library construction. Finally, the two samples were sequenced through Illumina technology (MiSeq; Illumina, San Diego, CA, USA).

### Agilent SureSelect Whole-Exome Target Enrichment

The two DNA samples were also processed using Agilent SureSelectXT Human All Exon Kit (version V5+UTRs; Agilent Technologies, CA, USA), following the manufacturer’s protocol as previously described [8]. The two whole-exome DNA librairies were sequenced through Illumina technology (HiSeq2500).

### Agilent HaloPlex Target Enrichment

The two DNA samples were also processed using a custom Haloplex panel (Agilent Technologies), which was designed using Agilent’s online design tool to target the same regions that were targeted by the RainDance assay (please see above). The total amplicon number was 9,991 and the target size was 230.7 kb with a theoretical coverage of 99.2% for our targeted regions. Target enrichment was performed according to the manufacturer's protocol for Illumina Sequencing (Version D.5; Agilent Technologies). The two DNA samples were split into 8 different reactions, each containing two restriction enzymes. Subsequently, all reactions were pooled back into one single tube, resulting in a digested DNA sample containing both targeted and non-targeted regions of the genome. The DNA sample was mixed with probes and hybridized overnight. Each end of the probe was complementary to a part of the single-stranded DNA fragments, and the center part was double stranded and comprised a cassette with the Illumina sequence motifs. Thousands of probes targeting different regions were added in this step, guiding circularization of the targeted fragments. The probes contained biotin, enabling the capture on streptavidin coated paramagnetic beads. Using a magnet rack, the targeted fragments were captured along with probes whereas other parts of the genome were discarded by removing the supernatant. Fragments correctly hybridized to their corresponding probe formed templates for a DNA ligase. Ligation resulted in the formation of closed DNA circles of targeted fragments. The targeted fragments were subsequently amplified by PCR. Sequence motifs needed for Illumina sequencing were associated, including sample barcodes which allowed samples to be pooled after this step. The samples were sequenced through Illumina technology (MiSeq).

### Nextera Rapid Capture Expanded Exome

The two DNA samples were lastly processed using Nextera Rapid Capture Expanded Exome (Illumina), applying pre-capture multiplexing, according to the manufacturer's protocol. A tagmentation was performed with the Nextera transposome (Illumina), which fragments the genomic DNA and adds adapter sequences allowing amplification by PCR. The process of enrichment involved two hybridizations so as to increase the specificity of the captured regions. A second PCR amplification step increased the amount of enriched DNA library for sequencing. The two samples were sequenced through Illumina technology (HiSeq2500).

### Sanger sequencing

The 10 mutations that were detected by only one or two enrichment technologies were investigated by a standard Sanger sequencing. Primer sequences and PCR conditions are available upon request. Amplified fragments were bidirectionally sequenced and analyzed using the automated 3730xl DNA Analyzer (Applied Biosystems, Waltham, MA, USA). Electrophoregram reads were assembled and read using Variant Reporter software (Applied Biosystems).

### Data Analysis

Demultiplexing of sequence data were performed with CASAVA (version 1.8.2). Subsequently, sequence reads from FASTQ files were mapped to the human genome (hg19/GRC37) using the Burrows-Wheeler Aligner (version 0.6.1; algorithm "BWA-SW"; default parameters) for targeted sequencing data, and CASAVA software (version 1.8.2, Illumina) for whole exome sequencing (WES) data. Variant calling was performed with SAMtools (version 0.1.18; algorithm "pileup" with the following parameters:-u -g -B -m 3 -C 50 -d 1000000 -L 1000000 -F 0.0002 -Q 0) for targeted sequencing data, and CASAVA software for WES data. The output VCF files were filtered using quality threshold of read depth ≥ 8×. All detected variants were annotated using the Ensembl Perl API (version 71) and other Perl scripts to include data from dbSNP and dbNSFP. The coverage for each targeted region as well as other quality control statistics was obtained by in-house Perl scripts and Bedtools 2.17. Through the four enrichment technologies (RainDance, Agilent SureSelect, Agilent HaloPlex and Nextera) in combination with Illumina sequencing, we always obtained a mean read depth higher than 100× for both samples.

Importantly, so as to avoid any biases, we only analyzed the intersection of the four BED files (related to RainDance, Agilent SureSelect, Agilent HaloPlex and Nextera), which included coding exons and UTRs with 10 bp of intronic flanking regions, of the 43 genes involved in monogenic forms of diabetes and obesity. Therefore, the analysis was restricted to the regions covered in theory by all the enrichment methods.

## Results

**Fig 1:**
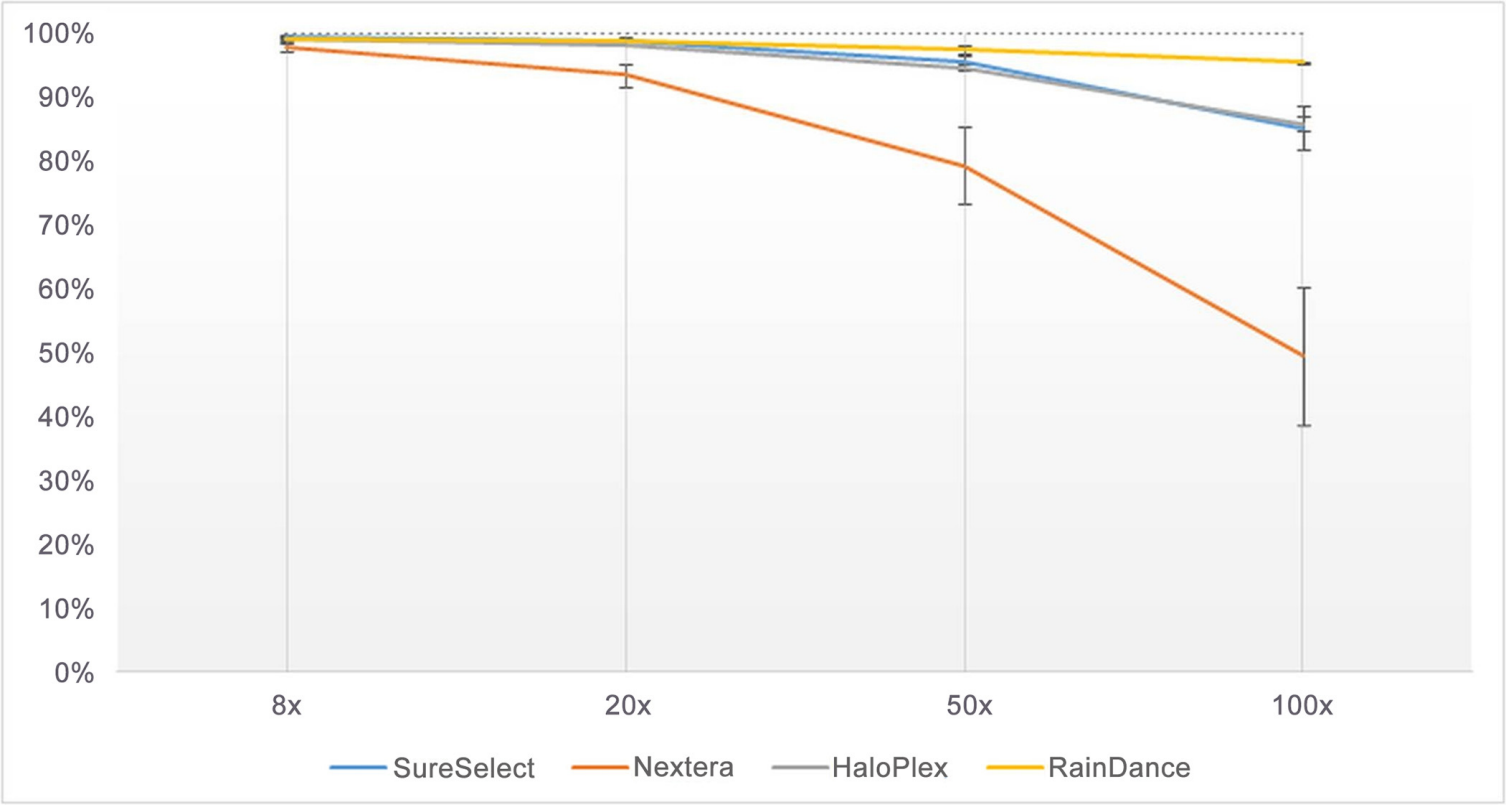
Percentage of base-pairs covered by 8, 20, 50 or 100 sequence reads in coding regions of targeted genes (with 10 bp of intronic flanking regions), according to each enrichment method.

We first compared the mean percentage of base-pairs covered by 8, 20, 50 or 100 sequence reads in coding regions (with 10 bp of intronic flanking regions) of the 43 targeted genes, according to each enrichment method (**Fig 1**). The mean percentage of base-pairs covered by 8 reads was quite similar between the four methods (from 97.9% for Nextera to 99.3% for SureSelect; **Fig 1**). However, when we consider a read depth ranging from 20 to 100×, only the RainDance enrichment method led to a good quality of sequencing coverage, with a mean percentage of covered base-pairs higher than 95% (**Fig 1**). The SureSelect and Haloplex methods led to a similar sequencing quality with a mean percentage of covered base-pairs of ~85%, at read depth of 100× (**Fig 1**). The Nextera method led to a poor sequencing quality right from a read depth of 20× (**Fig 1**).

**Fig 2:**
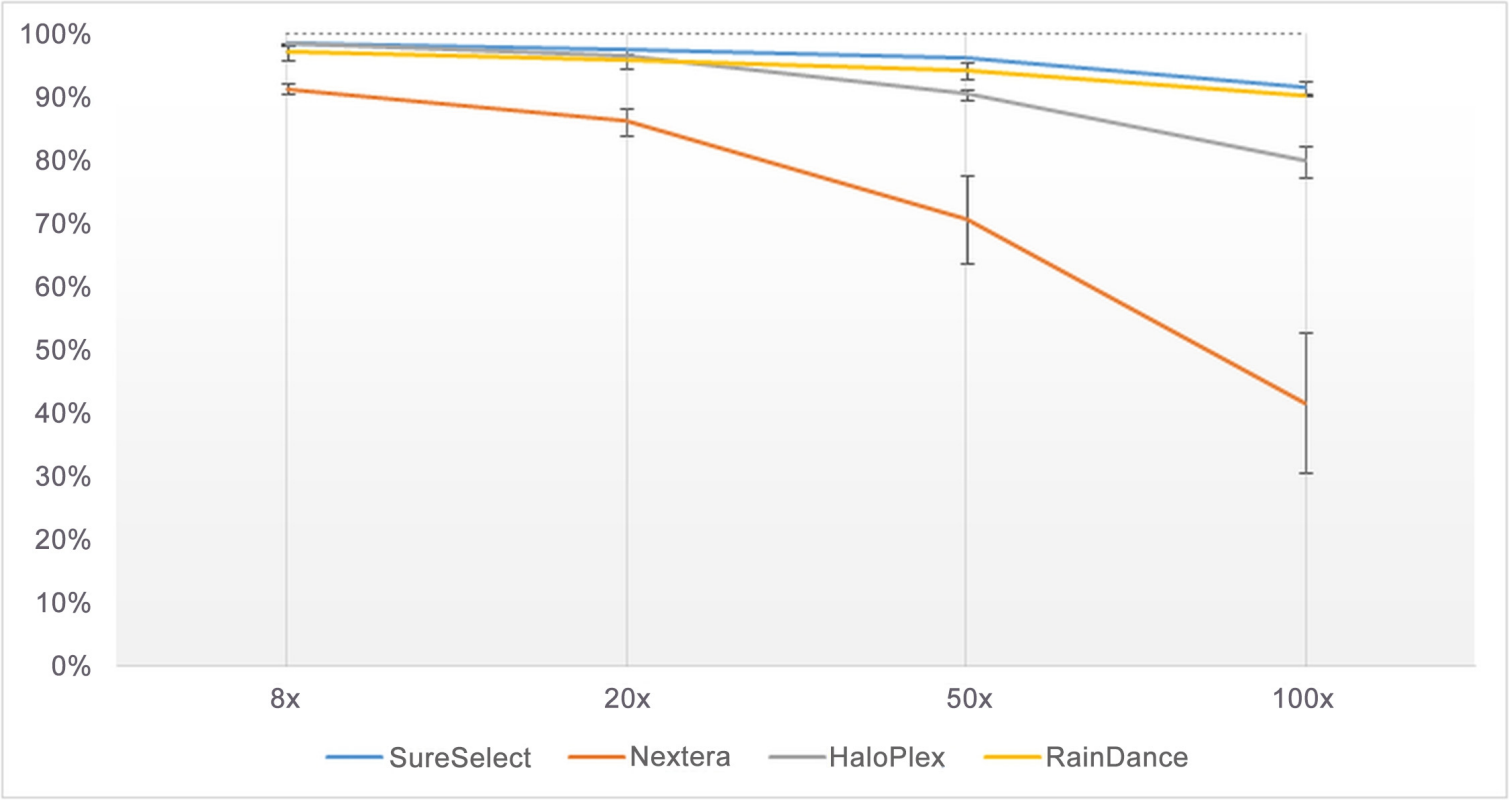
Percentage of base-pairs covered by 8, 20, 50 or 100 sequence reads in UTRs of targeted genes, according to each enrichment method.

Subsequently, using the same method, we analyzed the sequencing quality of UTRs separately as mutations in UTRs do not lead to highly straightforward interpretation, although they may be important for studying possible disruption of binding sites of both transcription factors in 5’UTRs and miRNAs in 3’UTRs. Sequencing qualities resulting from the RainDance and SureSelect enrichment methods were both quite good, with a mean percentage of covered base-pairs higher than 90%, at read depth of 100× (**Fig 2**). Sequencing qualities resulting from the HaloPlex and Nextera enrichment methods were poorer, with a mean percentage of covered base-pairs of 79.7% and 41.6% respectively, at read depth of 100× (**Fig 2**).

**Fig 3:**
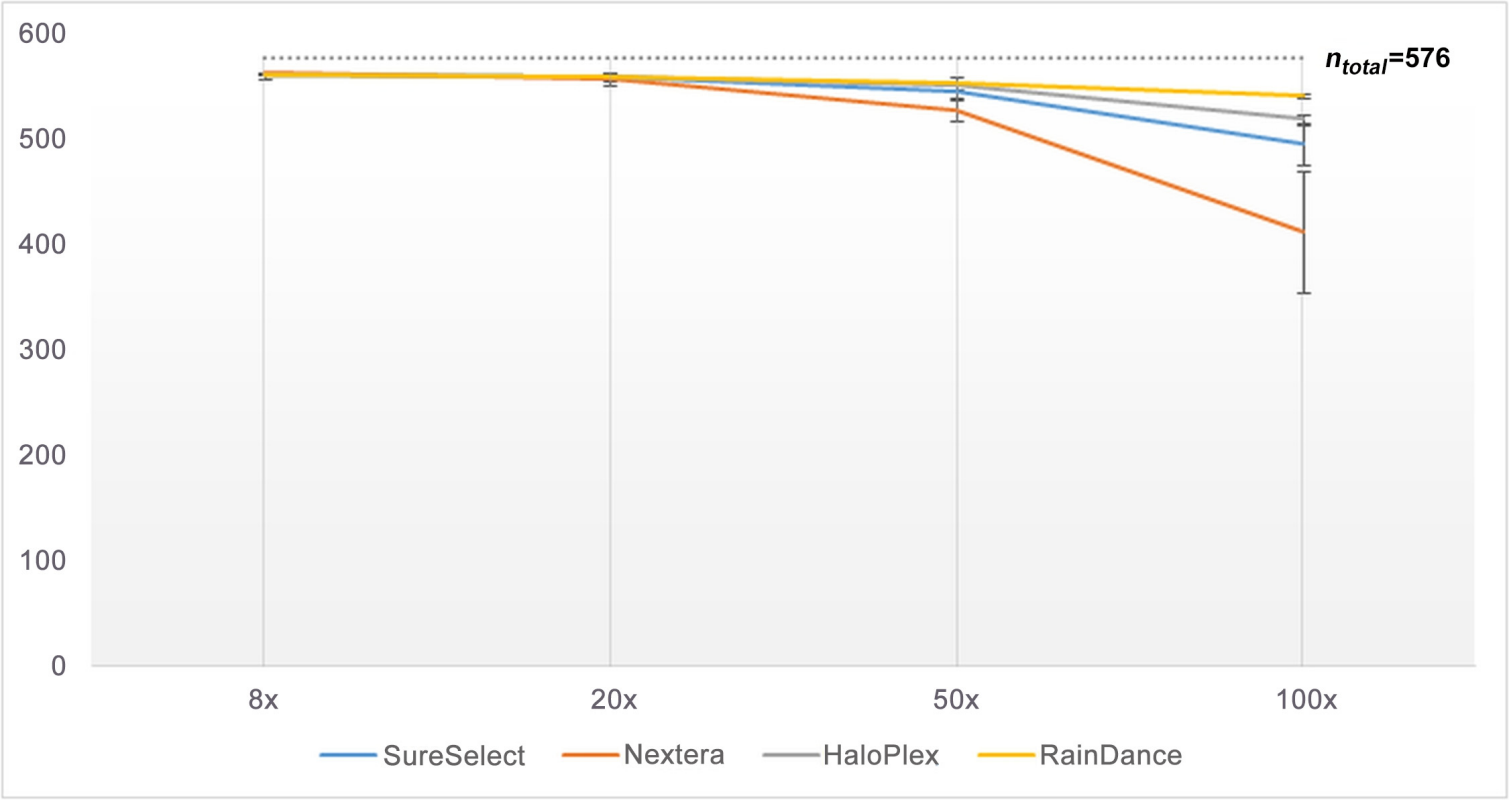
Number of coding exons (with 10 bp of intronic flanking regions; *n*_*total*_ = 576) exhaustively covered by 8, 20, 50 or 100 sequence reads, according to each enrichment method.

**Fig 4:**
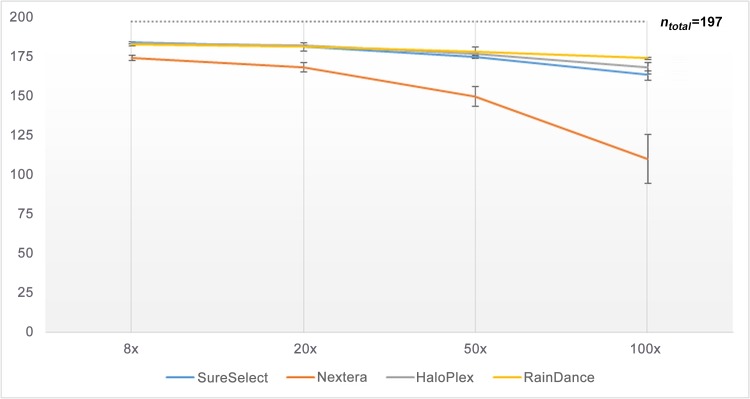
Number of UTRs (*n*_*total*_ = 197) exhaustively covered by 8, 20, 50 or 100 sequence reads, according to each enrichment method.

Subsequently, we compared the number of regions (coding exons or UTRs) fully covered according to each enrichment method, at read depth of 8×, 20×, 50× or 100× (**Figs 3**and **4**); so as to know if the missing base-pairs were uniformly distributed in the target or were present at specific loci (which would facilitate an eventual resequencing). The profile of covered coding regions (**Fig 3**) and covered UTRs (**Fig 4**) at the different read depths (8×, 20×, 50× or 100×) was in fact very similar to what was observed in **Figs 1**and **2**respectively (with a correlation [r²] higher than 0.9). Taken as a whole, the number of fully covered regions was higher for RainDance, slightly lower for SureSelect and HaloPlex, and much lower for Nextera (**Figs 3**and **4**). For a clinical diagnosis purpose, few regions had to be resequenced when having used RainDance, SureSelect or HaloPlex enrichment methods; but a lot more regions needed to be reassessed when having used the Nextera method.

**Fig 5:**
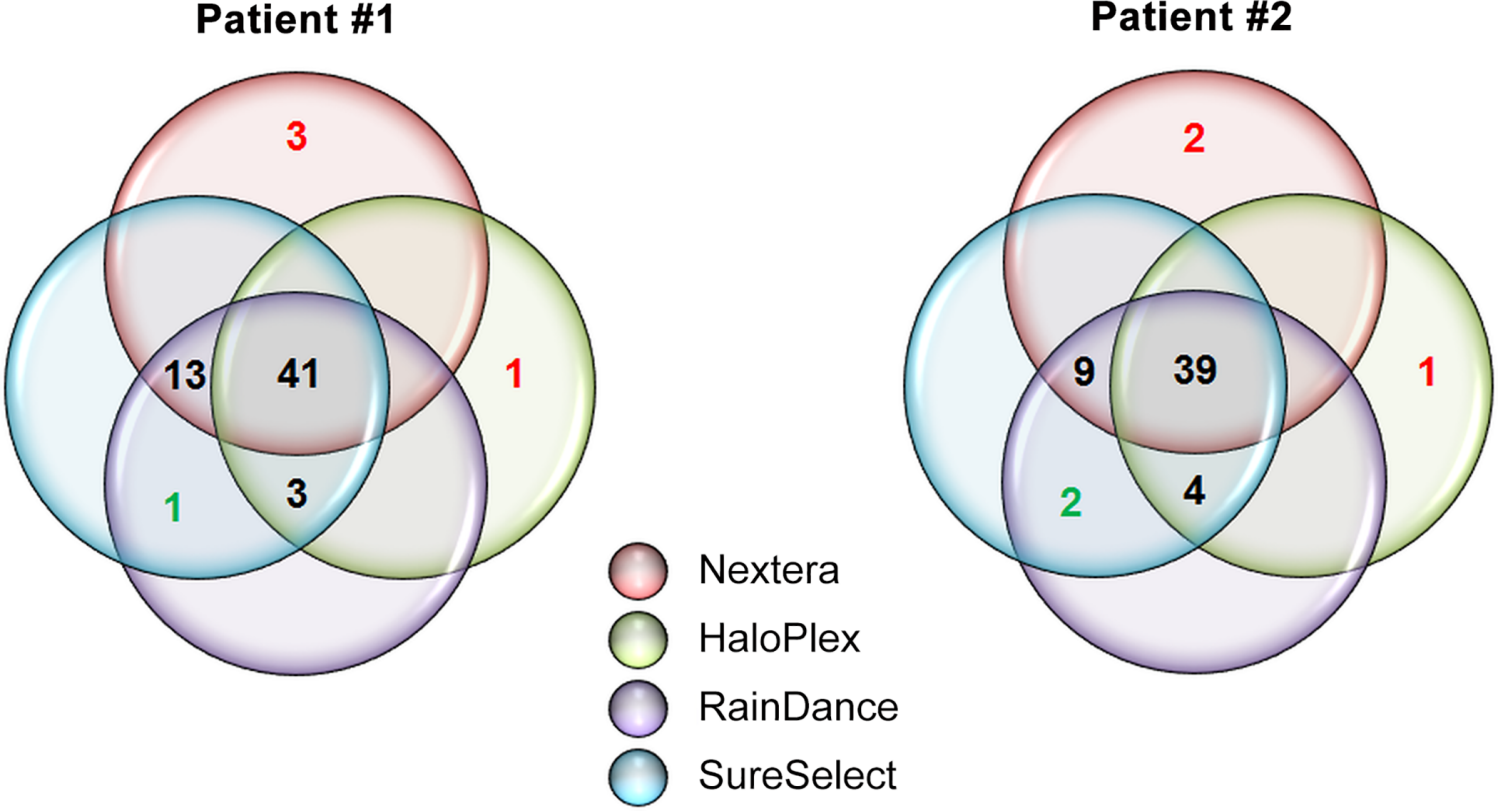
Venn diagram displaying the total number of variants in the targeted genes (coding exons and UTRs, with 10 bp of intronic flanking regions), which were identified by each enrichment method, in Patient #1 and Patient #2. The presence of mutations colored in red was not confirmed by Sanger sequencing while the presence of mutations colored in green was confirmed by Sanger sequencing (see also **S1 Fig**).

Subsequently, we compared the number of detected variants in the targeted genes (coding exons and UTRs, with 10 bp of intronic flanking regions) according to each enrichment method. When variants were detected by two different methods or less, we performed Sanger sequencing to validate or not the presence of these variants (**S1 Fig**). When variants were detected by at least three different methods, we considered that these variants were real. Unexpectedly, we found that the HaloPlex enrichment method led to high false negative rate (24% for Patient #1 and 20% for Patient #2; **Fig 5**), despite a good sequencing coverage (**Figs 1, 2, 3**and **4**). To a lesser extent, the Nextera enrichment method also missed some variants (7% for Patient #1 and 11% for Patient #2; **Fig 5**). Of note, the vast majority (> 90%) of the missing variants resulting from both HaloPlex and Nextera enrichment methods had an ID in dbSNP database (**S1 Table**). Moreover, these two enrichment methods led to false positive variants (from one to three variants in both patients; **Fig 5**). Of note, all these variants did not have any ID in dbSNP database (**S1 Table**) and the read depths at these loci were between 10 and 50× (**S1 Fig**). Nonetheless, it is noteworthy that all enrichment methods (including Nextera and Haloplex) did identify the mutation causing MODY in each patient (p.Gln382* in *HNF1B* for Patient #1 and p.His69_Lys71delinsArg in *HNF1B* for Patient #2; **S1 Table**).

## Discussion

In the present study, we compared four different enrichment methods including SureSelect, Nextera, HaloPlex and RainDance, in two patients presenting with MODY. The analyses were focused on genes (coding regions and UTRs) involved in monogenic forms of diabetes and obesity. We found that none of the methods led to full sequencing of the targets. Nonetheless, the RainDance, SureSelect and HaloPlex methods led to the best sequencing coverage of coding regions and UTRs; while the Nextera method led to a poor sequencing coverage. To get an exhaustive sequencing coverage of the target, there are several options:

1. to resequence the missing targets through Sanger sequencing, although it could be obviously labor-intensive and time-consuming;
2. to improve the low coverage in the missing targets through the combination of two NGS enrichment methods. For instance, Miya *et al*. have recently combined the SureSelect and HaloPlex methods in such a way that HaloPlex was used to target the protein-coding sequences with a read depth <15× in the SureSelect results [9].
3. to improve the low coverage in the missing targets by changing the design of amplicons targeting these low-covered regions (in the HaloPlex or RainDance libraries for instance) [7,10]. Indeed, the use of primers could lead to specificity issues through their hybridization at multiple loci of the genome or in cases where they target a locus containing a genetic variant.
4. to improve the low coverage in the missing targets by adding more probes (baits) targeting these low-covered regions (in the SureSelect libraries for instance). One company, Personalis, and two universities, Baylor College of Medicine and Emory Genetics Laboratory, are currently providing a ‘medical exome’ with an enhanced coverage for disease-associated genes, which improves the yield of molecular diagnosis of genetic diseases [11].

When we compared the detection of variants according to enrichment methods, we unexpectedly found that the HaloPlex method missed about 20% of variants, while the sequencing coverage was high. We also found some false negative variants through the use of Nextera. These missing variants may be explained by the use of enzymes by both HaloPlex and Nextera methods. Nextera strategy indeed relies on the tagmentation which is the use of a transposase to fragment DNA and to add adapters for multiplexing and sequencing. This transposase creates DNA fragments with a broad size range distribution, typically between 300-1000bp. The first issue is that all the DNA fragments are larger than the sequencing reads. Therefore, even with an enrichment step, some of the DNA fragments will be sequenced targeting regions of interest but also an unintended part that could be important depending on the size of the fragment. This phenomenon creates off-target in the sequencing data which results in a lower coverage for the same amount of library as a SureSelect library for instance. The second issue involves the concentration of input genomic DNA which has to be very accurate and proportional to the quantity of transposomes, otherwise the size distribution of the library will not be in the expected range. The third issue of transposome is a bias towards target regions with high GC content [12]. All these reasons could explain false positive/negative rates but it may also be due to other critical steps of library preparation like the number of PCR cycles and purification steps. However, we have to acknowledge that one of the limitations of our study is the use of different Illumina sequencers (MiSeq and HiSeq), even if we used the same chemistry (flowcell, cluster generation process, paired-end reads). Moreover, we analyzed two DNA samples only, which is another limitation. Further studies are needed to confirm the present results.

The question of which NGS technique yields the highest diagnosis rate is frequently discussed in the literature and is still open. It has been demonstrated that whole-genome sequencing (WGS) was more appropriate as copy number variants (CNVs) can be detected more accurately [13]. Some other groups have shown that whole-exome sequencing (WES) was a good alternative to WGS as it costs three to four times less and the results in term of variants are very similar [14]. Finally, some panel-based genetic testing studies, including our present study, have been demonstrated to lead to better results than WES with a higher sensitivity and specificity [15]. Actually, the answer relative to the best NGS method for genetic testing may depend on the studied disease itself: a disease which is known to involve CNVs or big structural changes like severe intellectual disability and autism will require WGS. However, WES is still cost-effective for many diseases and may require technical modifications to get a good coverage for the genes specific to the disease of interest. Finally, target sequencing is still in many cases the best method as it is very accurate, cost-effective, and is much quicker and easier to interpret as the number of genes is limited and there are not any incidental findings to report. Importantly, we have shown here that the use of enzymes to fragment DNA (either in target sequencing [HaloPlex] or WES [Nextera]) might be not the best strategy to get an accurate sequencing.

## Supporting Information

S1 Fig Sanger sequencing of mutations detected by only one or two enrichment technologies in Patients #1 and #2. (DOCX)

S1 Table List of variants in the targeted genes (coding exons and UTRs, with 10 bp of intronic flanking regions), which were identified by each enrichment method, in Patients #1 and #2. (DOCX)
