# Supplementary material for: What Is the Best NGS Enrichment Method for the Molecular Diagnosis of Monogenic Diabetes and Obesity?
Source: PLoS One. 2015 Nov 23;10(11):e0143373. doi: 10.1371/journal.pone.0143373 (PMC4657897; doi:10.1371/journal.pone.0143373)
Supplement: S1 Fig — (DOCX) [file pone.0143373.s001.docx]

**Supplementary Figure 1. Sanger sequencing of mutations detected by only one or two enrichment technologies in Patients #1 and #2**

**
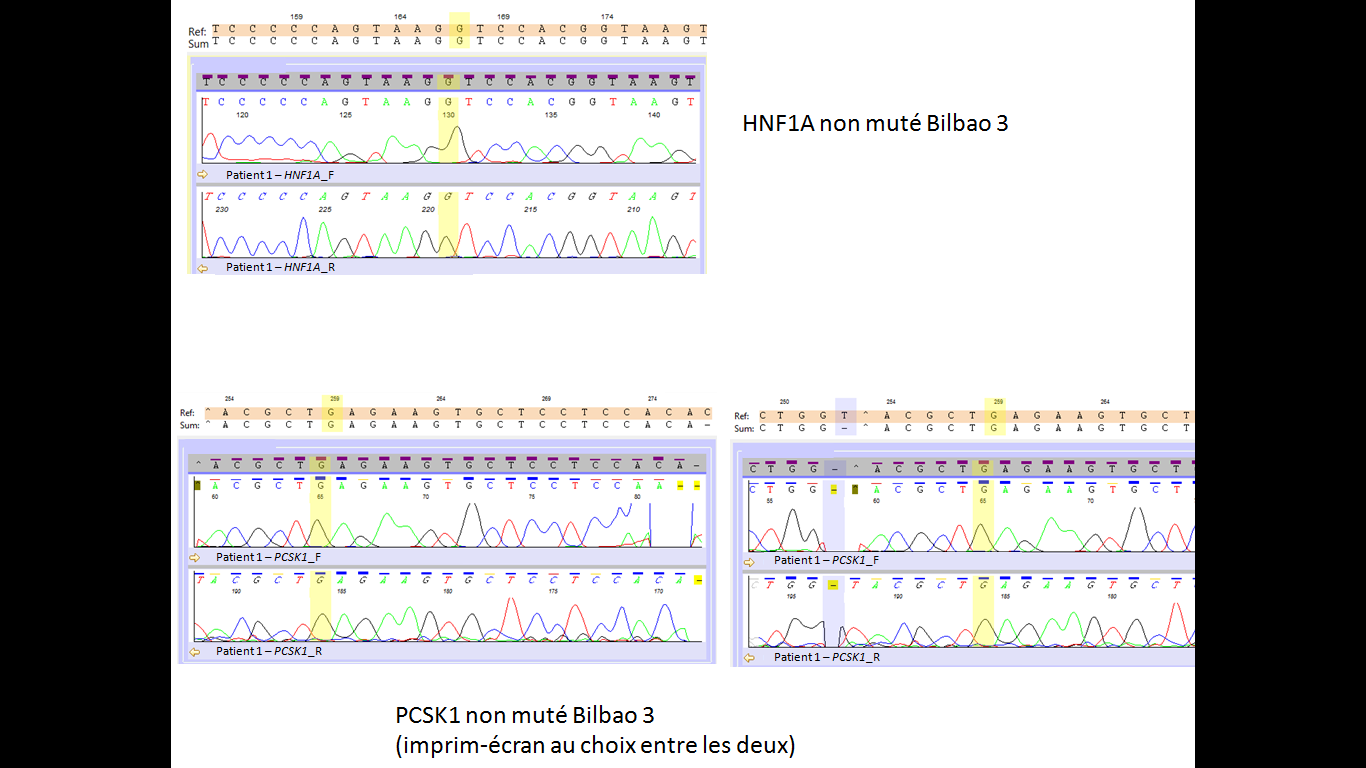
Patient #1**

*HNF1A* p.Lys316Asn => not confirmed (highlighted in yellow) [primarily found by Nextera at a depth of 21×]

*
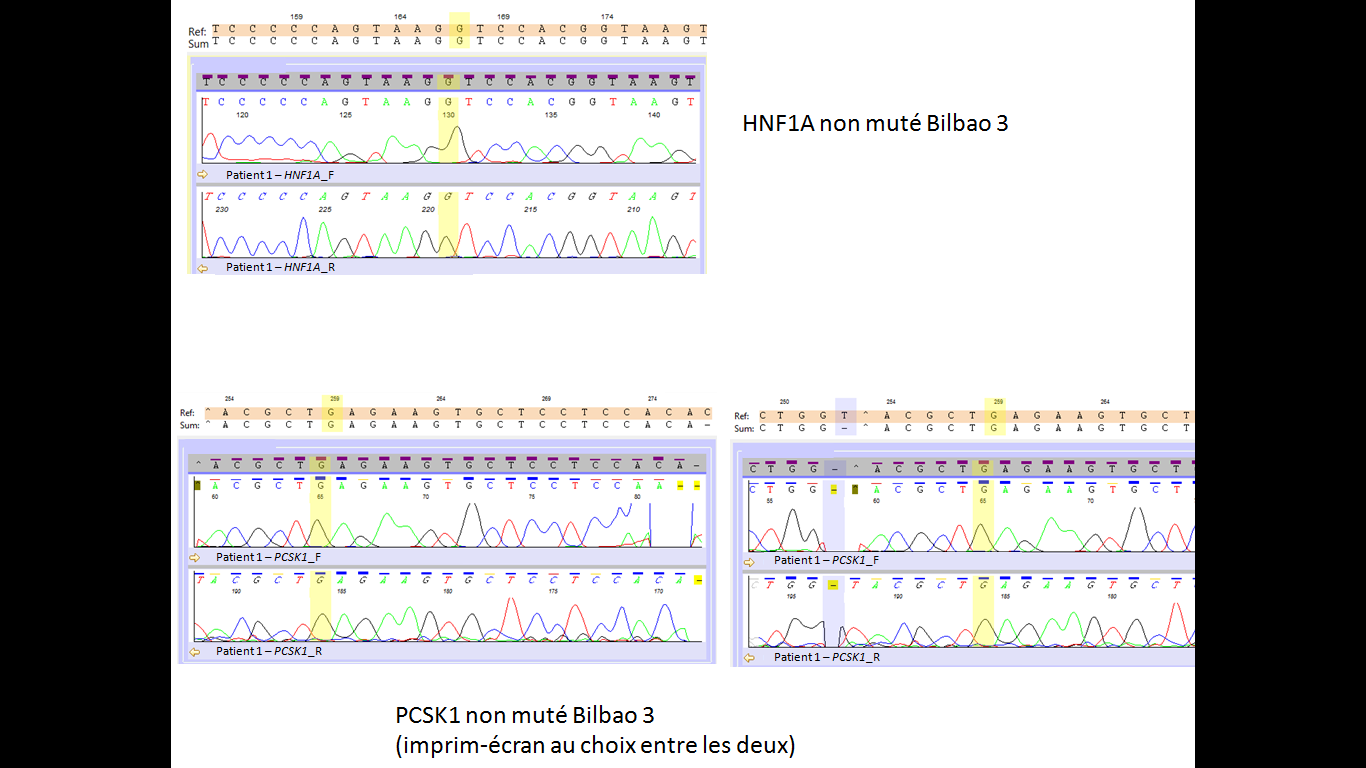
*

*PCSK1* p.Ala344_Leu351delins8 => not confirmed (highlighted in yellow) [primarily found by Nextera at a depth of 37×]

*
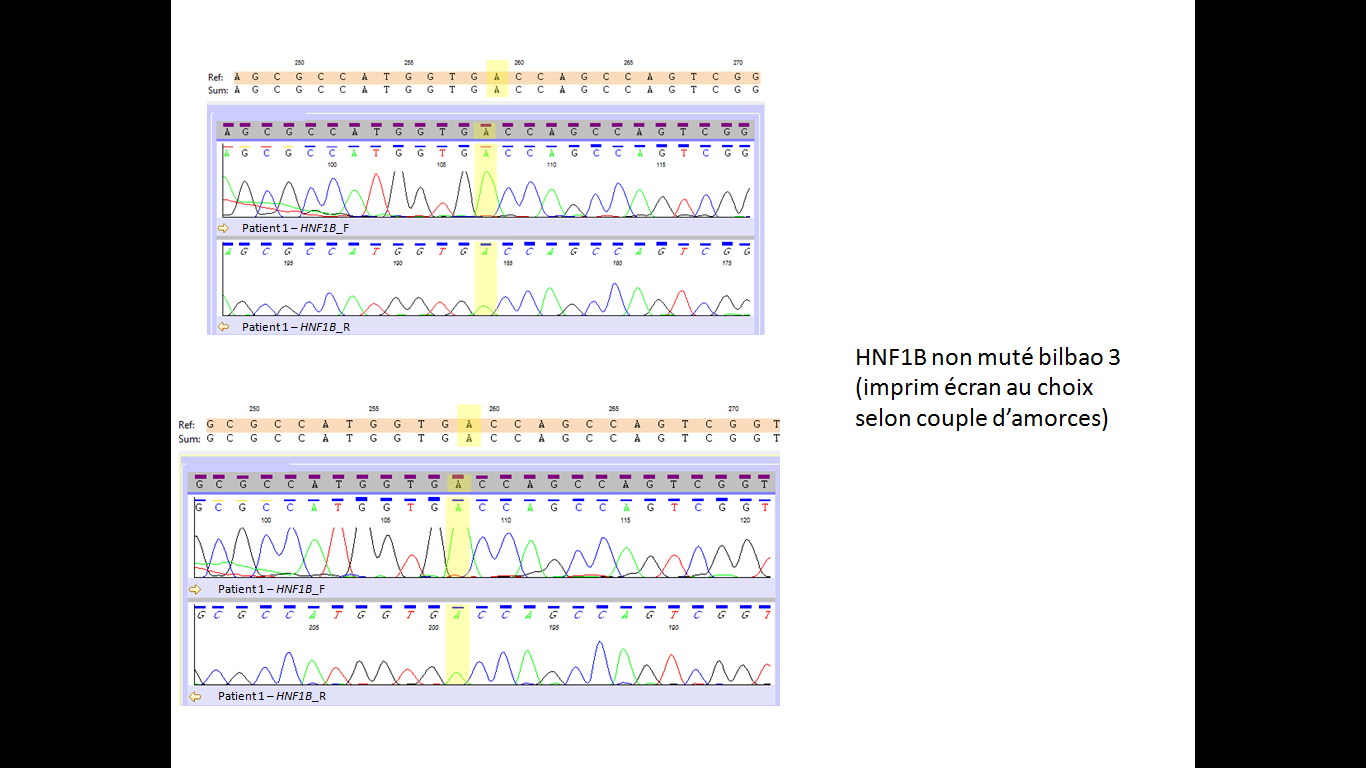
*

*HNF1B* p.Thr376Pro => not confirmed (locus highlighted in yellow) [primarily found by HaloPlex at a depth of 37×]

*
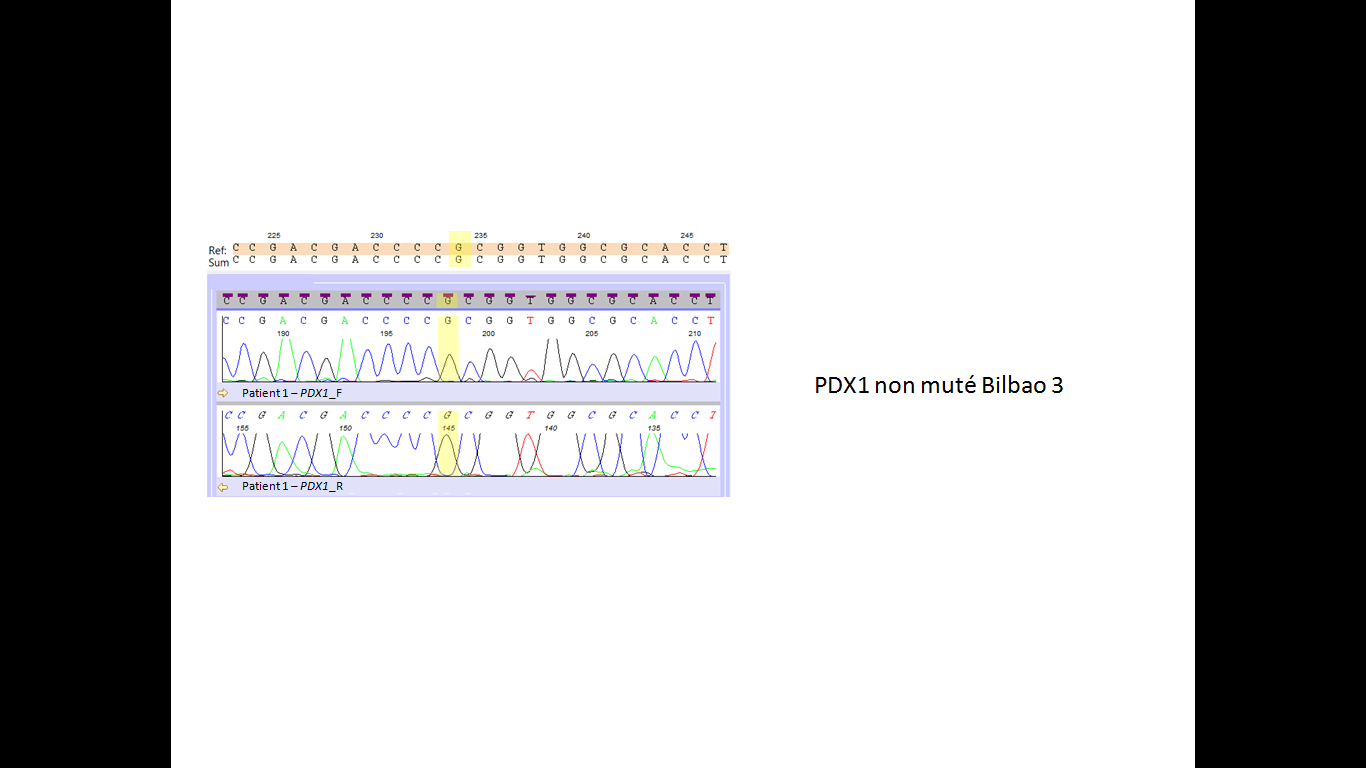
*

*PDX1* p.Ala78Glu => not confirmed (locus highlighted in yellow) [primarily found by Nextera at a depth of 14×]

*
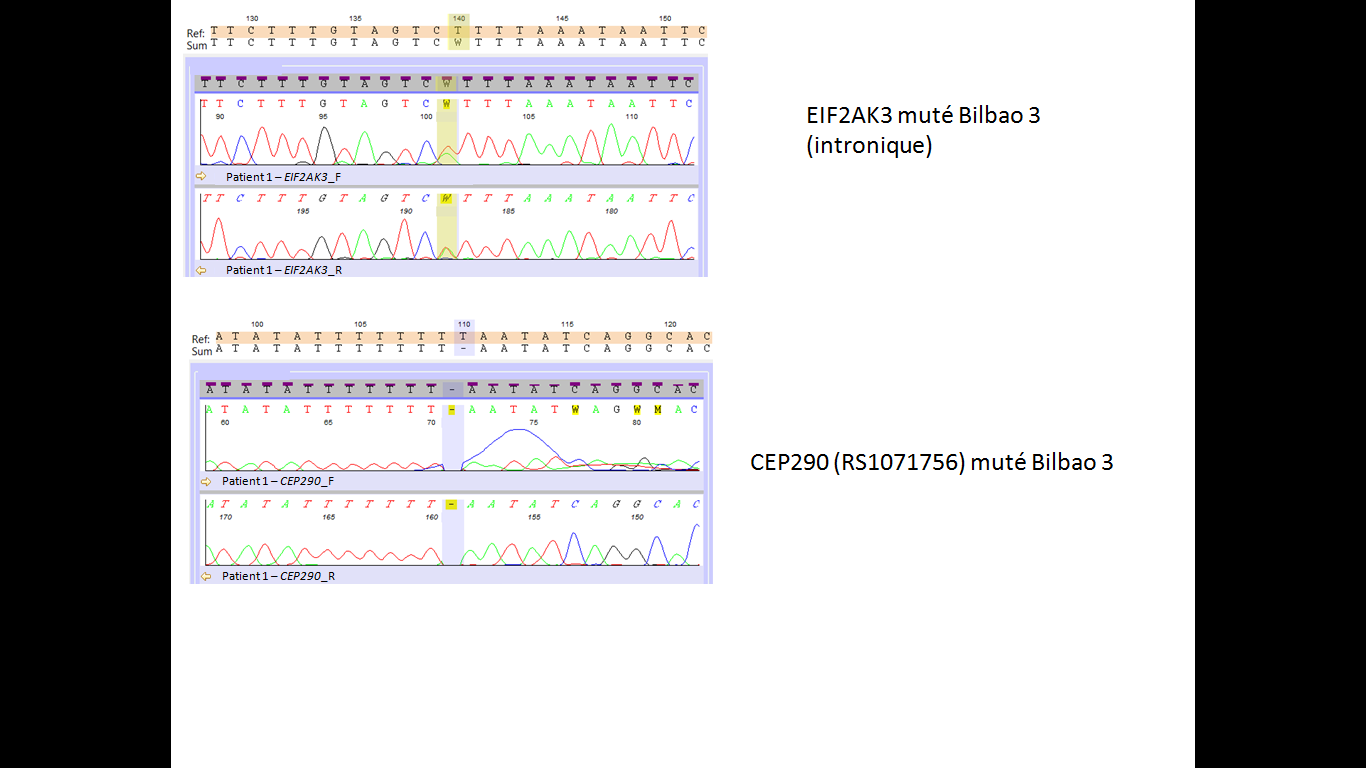
CEP290* c.3574-9del => confirmed (locus highlighted in blue) [primarily found by SureSelect and RainDance at a depth of 282× and 201×, respectively]

**
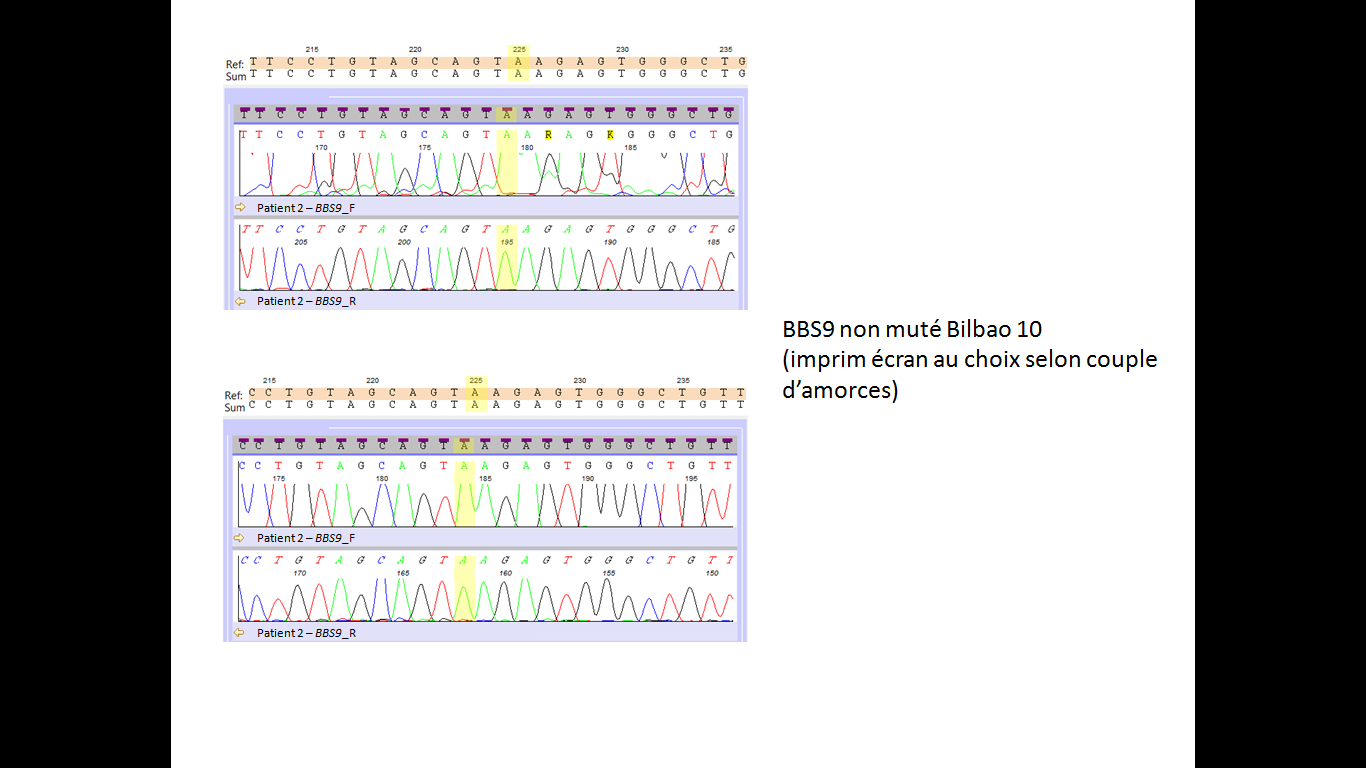
Patient #2**

*BBS9* c.999A>G => not confirmed (locus highlighted in yellow) [primarily found by HaloPlex at a depth of 10×]

*
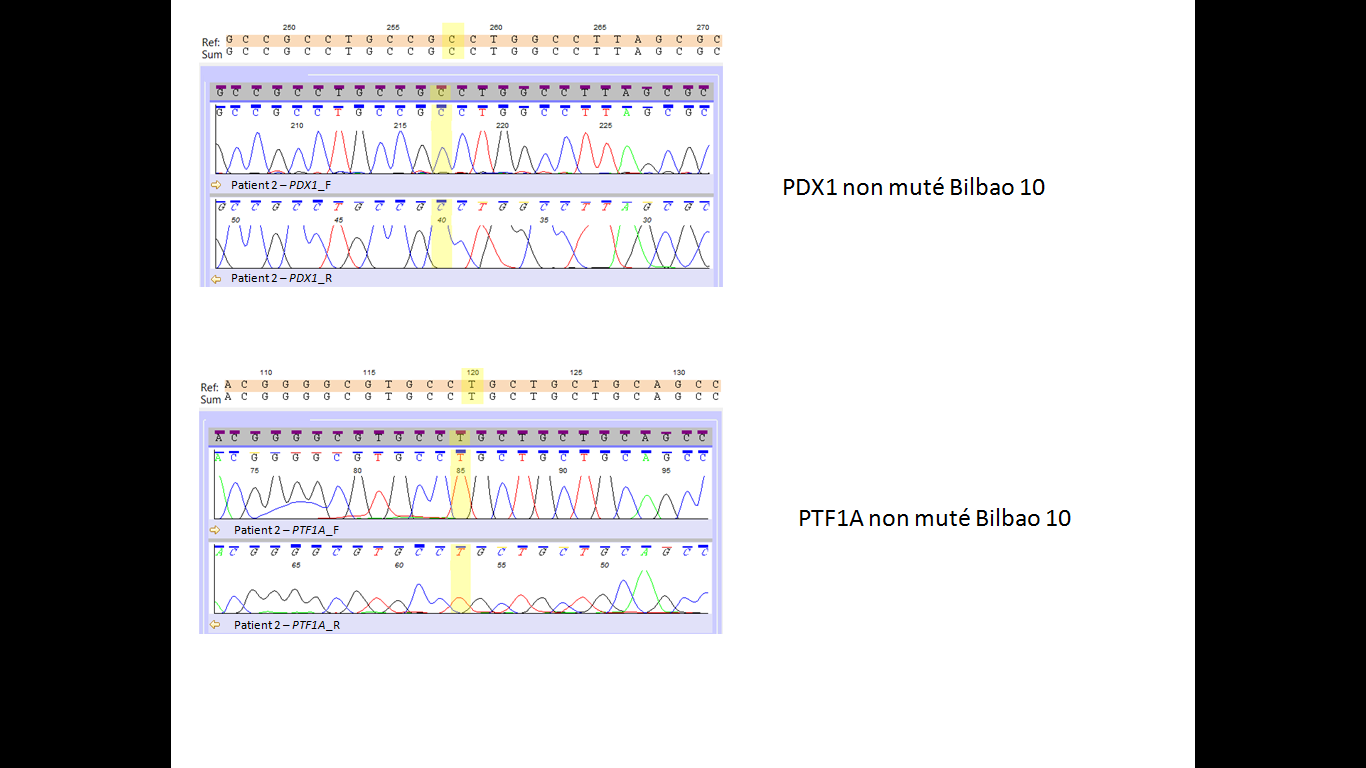
*

*PDX1* p.Pro263His => not confirmed (locus highlighted in yellow) [primarily found by Nextera at a depth of 15×]

*
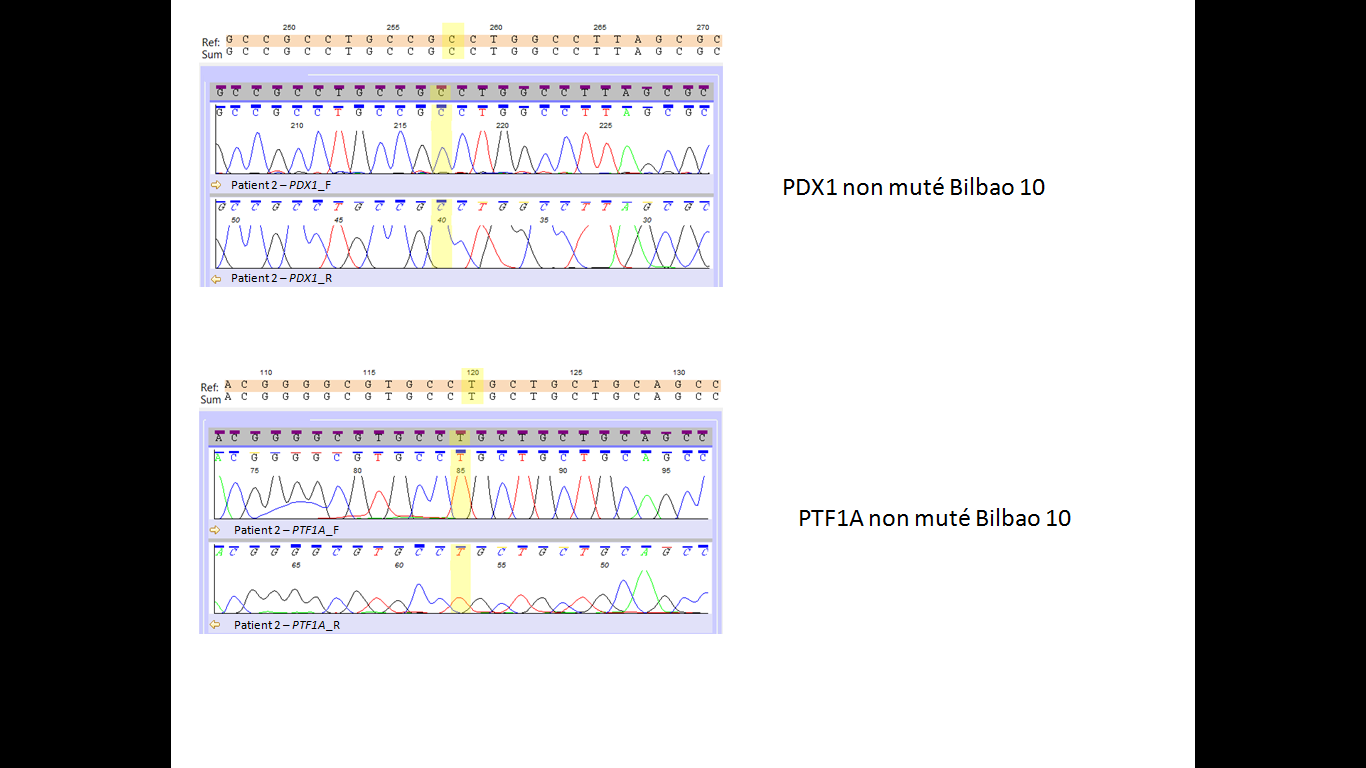
*

*PTF1A* p.Leu68Pro => not confirmed (locus highlighted in yellow) [primarily found by Nextera at a depth of 10×]

*
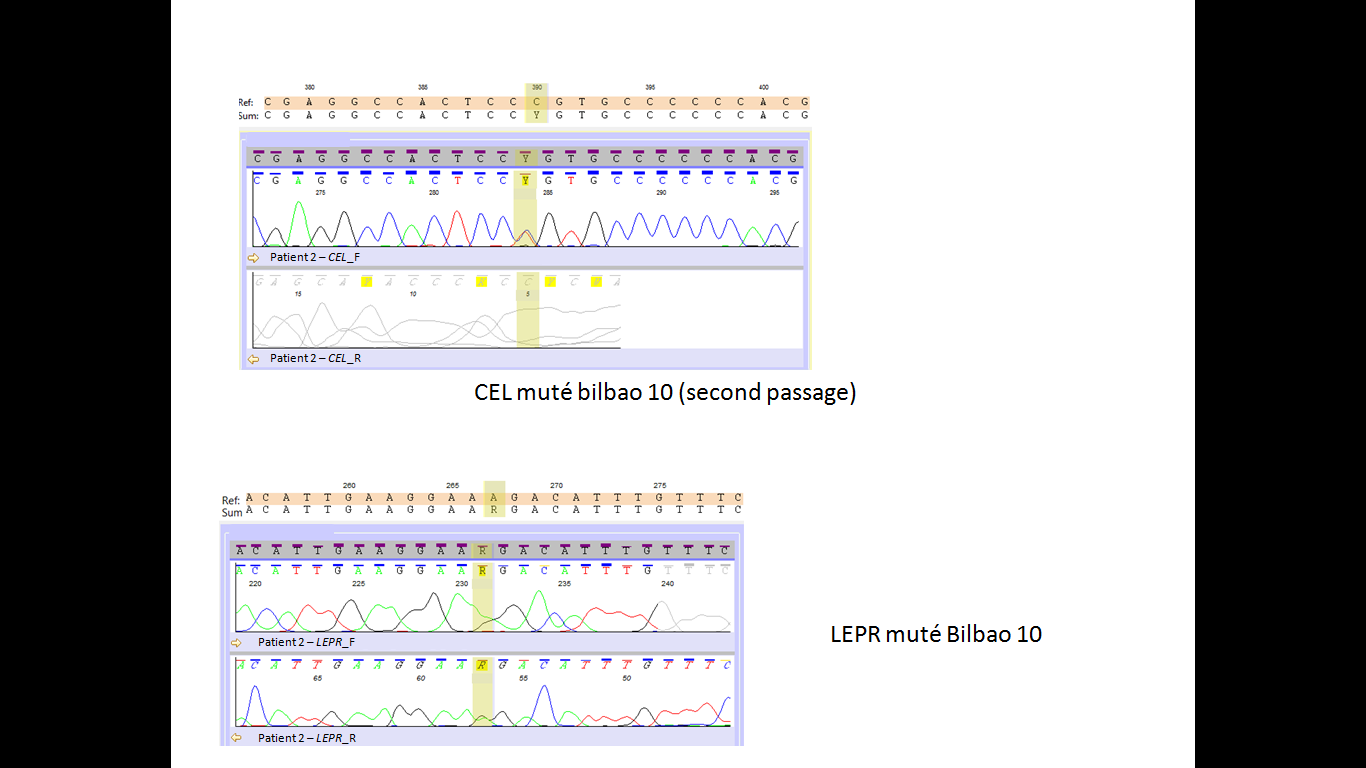
*

*CEL* rs488087 => confirmed (locus highlighted in yellow) [primarily found by SureSelect and RainDance at a depth of 26× and 130×, respectively]

*
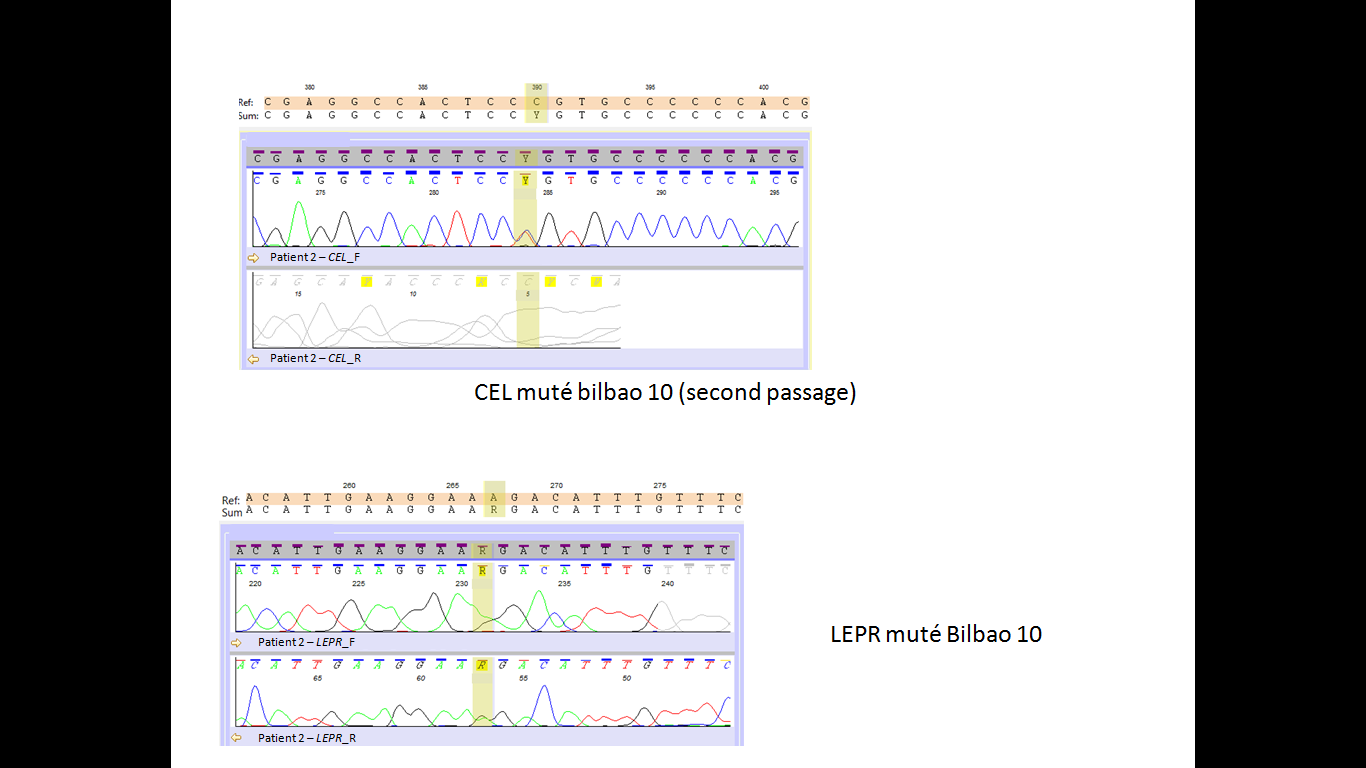
LEPR* p.Lys109Arg => confirmed (locus highlighted in yellow) [primarily found by SureSelect and RainDance at a depth of 218× and 241×, respectively]
