# Supplementary material for: What Is the Best NGS Enrichment Method for the Molecular Diagnosis of Monogenic Diabetes and Obesity?
Source: PLoS One. 2015 Nov 23;10(11):e0143373. doi: 10.1371/journal.pone.0143373 (PMC4657897; doi:10.1371/journal.pone.0143373)
Supplement: S1 Table — (DOCX) [file pone.0143373.s002.docx]

**Supplementary Table 1. List of variants in the targeted genes (coding exons and UTRs, with 10 bp of intronic flanking regions), which were identified by each enrichment method, in Patients #1 and #2**

| **Patient** | **Gene** | **Variant Type** | **Variant Location** | **Coding Effect** | **cNomenclature** | **pNomenclature** | **rsID** | **rsMAF** | **Sure-Select** | **Next-era** | **Halo-Plex** | **Rain-Dance** |
| --- | --- | --- | --- | --- | --- | --- | --- | --- | --- | --- | --- | --- |
| #1 | *ABCC8* | Subs. | Exon | Missense | NM_001287174.1:c.4108G>T | p.Ala1370Ser | rs757110 | 0.29 | Yes | Yes | Yes | Yes |
| #1 | *ABCC8* | Subs. | Intron | No | NM_001287174.1:c.2117-3C>T | p.? | rs1799854 | 0.42 | Yes | Yes | Yes | Yes |
| #1 | *ABCC8* | Subs. | Exon | Synonymous | NM_001287174.1:c.207T>C | p.= | NA | NA | Yes | Yes | Yes | Yes |
| #1 | *BBS1* | Subs. | Exon | Synonymous | NM_024649.4:c.378G>A | p.= | rs2298806 | 0.23 | Yes | Yes | Yes | Yes |
| #1 | *BBS1* | Subs. | Intron | No | NM_024649.4:c.724-8G>C | p.? | rs10896125 | 0.25 | Yes | Yes | Yes | Yes |
| #1 | *BBS1* | Subs. | Exon | Synonymous | NM_024649.4:c.1413C>T | p.= | rs3816492 | 0.17 | Yes | Yes | Yes | Yes |
| #1 | *BBS1* | Subs. | 3'UTR | No | NM_024649.4:c.*7A>G | p.? | rs8432 | 0.35 | Yes | Yes | Yes | Yes |
| #1 | *BBS12* | Subs. | Exon | Missense | NM_152618.2:c.1157G>A | p.Arg386Gln | rs309370 | 0.48 | Yes | Yes | No | Yes |
| #1 | *BBS12* | Subs. | Exon | Synonymous | NM_152618.2:c.1398C>T | p.= | rs2292493 | 0.27 | Yes | Yes | Yes | Yes |
| #1 | *BBS2* | Subs. | Exon | Synonymous | NM_031885.3:c.209G>A | p.= | rs4784677 | 0.004 | Yes | Yes | No | Yes |
| #1 | *BBS4* | Subs. | Intron | No | NM_033028.4:c.77-6G>A | p.? | rs8033604 | 0.05 | Yes | Yes | Yes | Yes |
| #1 | *BBS4* | Subs. | Exon | Synonymous | NM_033028.4:c.906T>C | p.= | rs12914333 | 0.03 | Yes | Yes | Yes | Yes |
| #1 | *BBS4* | Subs. | Exon | Missense | NM_033028.4:c.1061T>C | p.Ile354Thr | rs2277598 | 0.45 | Yes | Yes | Yes | Yes |
| #1 | *BBS9* | Subs. | Exon | Missense | NM_198428.2:c.1993C>T | p.Leu665Phe | rs116262072 | 0.004 | Yes | Yes | Yes | Yes |
| #1 | *BDNF* | Subs. | Exon | Synonymous | NM_170735.5:c.450A>G | p.= | rs2353512 | 0.005 | Yes | Yes | Yes | Yes |
| #1 | *CEL* | Subs. | Exon | Synonymous | NM_001807.4:c.1719C>T | p.= | rs488087 | 0.24 | Yes | Yes | No | Yes |
| #1 | *CEP290* | Indel | Intron | No | NM_025114.3:c.3574-9del | p.? | rs10717563 | NA | Yes | No | No | Yes |
| #1 | *CEP290* | Subs. | Exon | Synonymous | NM_025114.3:c.2268A>G | p.= | rs2468255 | 0.2 | Yes | Yes | No | Yes |
| #1 | *EIF2AK3* | Indel | Exon | Missense | NM_004836.5:c.61_63delins--- | p.Leu21 | NA | NA | Yes | No | Yes | Yes |
| #1 | *EIF2AK3* | Subs. | Exon | Missense | NM_004836.5:c.2110G>T | p.Ala704Ser | rs1805165 | 0.29 | Yes | Yes | Yes | Yes |
| #1 | *EIF2AK3* | Subs. | Exon | Missense | NM_004836.5:c.497A>G | p.Gln166Arg | rs13045 | 0.35 | Yes | Yes | Yes | Yes |
| #1 | *EIF2AK3* | Subs. | Exon | Missense | NM_004836.5:c.407C>G | p.Ser136Cys | rs867529 | 0.29 | Yes | Yes | Yes | Yes |
| #1 | *FOXP3* | Subs. | Exon | Synonymous | NM_014009.3:c.543C>T | p.= | rs2232367 | 0.02 | Yes | Yes | Yes | Yes |
| #1 | *GLIS3* | Subs. | Exon | Missense | NM_152629.3:c.902C>A | p.Pro301Gln | rs6415788 | 0.31 | Yes | No | Yes | Yes |
| #1 | *GLIS3* | Subs. | Exon | Missense | NM_152629.3:c.805T>C | p.Ser269Pro | rs806052 | 0.002 | Yes | No | Yes | Yes |
| #1 | *HNF1A* | Subs. | Exon | Synonymous | NM_000545.5:c.51C>G | p.= | rs1169289 | 0.41 | Yes | Yes | Yes | Yes |
| #1 | *HNF1A* | Subs. | Exon | Synonymous | NM_000545.5:c.864G>C | p.= | rs56348580 | 0.21 | Yes | Yes | Yes | Yes |
| #1 | *HNF1A* | Subs. | Intron | No | NM_000545.5:c.1501+7G>A | p.? | rs2464195 | 0.37 | Yes | Yes | Yes | Yes |
| #1 | *HNF1A* | Subs. | Exon | Synonymous | NM_000545.5:c.1545G>A | p.= | rs55834942 | 0.09 | Yes | Yes | Yes | Yes |
| #1 | *HNF1A* | Subs. | Exon | Synonymous | NM_000545.5:c.1720A>G | p.= | rs1169305 | 0.01 | Yes | Yes | Yes | Yes |
| #1 | *HNF1A* | Subs. | Exon | Missense | NM_000545.5:c.948G>T | p.Lys316Asn | NA | NA | No | Yes | No | No |
| #1 | *HNF1B* | Subs. | Exon | Nonsense | NM_000458.2:c.1144C>T | p.Gln382* | NA | NA | Yes | Yes | Yes | Yes |
| #1 | *HNF1B* | Subs. | Exon | Missense | NM_000458.2:c.1126A>C | p.Thr376Pro | NA | NA | No | No | Yes | No |
| #1 | *KCNJ11* | Subs. | Exon | Missense | NM_000525.3:c.1009G>A | p.Val337Ile | rs5215 | 0.28 | Yes | Yes | No | Yes |
| #1 | *KCNJ11* | Subs. | Exon | Missense | NM_000525.3:c.808C>G | p.Leu270Val | rs1800467 | 0.02 | Yes | Yes | Yes | Yes |
| #1 | *KCNJ11* | Subs. | Exon | Synonymous | NM_000525.3:c.570C>T | p.= | rs5218 | 0.28 | Yes | Yes | No | Yes |
| #1 | *KCNJ11* | Subs. | Exon | Missense | NM_000525.3:c.67A>G | p.Lys23Glu | rs5219 | 0.27 | Yes | Yes | No | Yes |
| #1 | *LEPR* | Subs. | Exon | Missense | NM_002303.5:c.326A>G | p.Lys109Arg | rs1137100 | 0.39 | Yes | Yes | No | Yes |
| #1 | *LEPR* | Subs. | Exon | Missense | NM_002303.5:c.668A>G | p.Gln223Arg | rs1137101 | 0.41 | Yes | Yes | Yes | Yes |
| #1 | *LEPR* | Subs. | Exon | Synonymous | NM_002303.5:c.1029T>C | p.= | rs1805134 | 0.19 | Yes | Yes | Yes | Yes |
| #1 | *LEPR* | Subs. | Exon | Synonymous | NM_002303.5:c.3057G>A | p.= | rs1805096 | 0.46 | Yes | Yes | Yes | Yes |
| #1 | *MKKS* | Subs. | Exon | Missense | NM_018848.3:c.1595G>T | p.Gly532Val | rs1545 | 0.17 | Yes | Yes | Yes | Yes |
| #1 | *MKKS* | Subs. | Exon | Missense | NM_018848.3:c.1549C>T | p.Arg517Cys | rs1547 | 0.17 | Yes | Yes | Yes | Yes |
| #1 | *MKKS* | Subs. | Exon | Synonymous | NM_018848.3:c.534C>T | p.= | rs17852625 | 0.17 | Yes | Yes | No | Yes |
| #1 | *MKKS* | Subs. | Exon | Synonymous | NM_018848.3:c.117C>T | p.= | rs16991547 | 0.2 | Yes | Yes | No | Yes |
| #1 | *NEUROG3* | Subs. | Exon | Missense | NM_020999.3:c.596T>C | p.Phe199Ser | rs4536103 | 0.45 | Yes | Yes | Yes | Yes |
| #1 | *NEUROG3* | Subs. | Exon | Missense | NM_020999.3:c.499G>A | p.Gly167Arg | rs41277236 | 0.03 | Yes | Yes | Yes | Yes |
| #1 | *PCSK1* | Indel | Exon | In-frame | NM_000439.4:c.1030_1051delins22 | p.Ala344_Leu351delins8 | NA | NA | No | Yes | No | No |
| #1 | *PCSK1* | Subs. | Exon | Missense | NM_000439.4:c.2069G>C | p.Ser690Thr | rs6235 | 0.25 | Yes | Yes | Yes | Yes |
| #1 | *PCSK1* | Subs. | Exon | Missense | NM_000439.4:c.1993C>G | p.Gln665Glu | rs6234 | 0.26 | Yes | Yes | Yes | Yes |
| #1 | *PCSK1* | Subs. | Exon | Synonymous | NM_000439.4:c.1650T>C | p.= | rs6233 | 0.34 | Yes | Yes | No | Yes |
| #1 | *PDX1* | Subs. | Exon | Missense | NM_000209.3:c.233C>A | p.Ala78Glu | NA | NA | No | Yes | No | No |
| #1 | *PTF1A* | Subs. | Exon | Missense | NM_178161.2:c.787T>C | p.Ser263Pro | rs7918487 | 0.38 | Yes | Yes | Yes | Yes |
| #1 | *SDCCAG8* | Subs. | Exon | Missense | NM_006642.3:c.1134A>T | p.Glu378Asp | NA | NA | Yes | Yes | Yes | Yes |
| #1 | *SDCCAG8* | Subs. | Exon | Synonymous | NM_006642.3:c.1725G>A | p.= | NA | NA | Yes | Yes | Yes | Yes |
| #1 | *WFS1* | Subs. | Intron | No | NM_006005.3:c.461-9A>G | p.? | rs10010131 | 0.27 | Yes | Yes | Yes | Yes |
| #1 | *WFS1* | Subs. | Exon | Synonymous | NM_006005.3:c.684C>G | p.= | rs1801213 | 0.24 | Yes | Yes | No | Yes |
| #1 | *WFS1* | Subs. | Exon | Missense | NM_006005.3:c.997G>A | p.Val333Ile | rs1801212 | 0.14 | Yes | Yes | Yes | Yes |
| #1 | *WFS1* | Subs. | Exon | Synonymous | NM_006005.3:c.1185C>T | p.= | rs1801206 | 0.33 | Yes | Yes | Yes | Yes |
| #1 | *WFS1* | Subs. | Exon | Synonymous | NM_006005.3:c.1500C>T | p.= | NA | NA | Yes | Yes | Yes | Yes |
| #1 | *WFS1* | Subs. | Exon | Synonymous | NM_006005.3:c.2433G>A | p.= | rs1046314 | 0.32 | Yes | Yes | Yes | Yes |
| #1 | *WFS1* | Subs. | Exon | Synonymous | NM_006005.3:c.2565A>G | p.= | NA | NA | Yes | Yes | No | Yes |
| #2 | *ABCC8* | Subs. | Intron | No | NM_001287174.1:c.2117-3C>T | p.? | rs1799854 | 0.42 | Yes | Yes | Yes | Yes |
| #2 | *ABCC8* | Subs. | Exon | Synonymous | NM_001287174.1:c.1686C>T | p.= | NA | NA | Yes | Yes | Yes | Yes |
| #2 | *ABCC8* | Subs. | Exon | Synonymous | NM_001287174.1:c.207T>C | p.= | NA | NA | Yes | Yes | Yes | Yes |
| #2 | *BBS1* | Subs. | 3'UTR | No | NM_024649.4:c.*7A>G | p.? | rs8432 | 0.35 | Yes | Yes | Yes | Yes |
| #2 | *BBS12* | Subs. | Exon | Missense | NM_152618.2:c.1157G>A | p.Arg386Gln | rs309370 | 0.48 | Yes | Yes | No | Yes |
| #2 | *BBS12* | Subs. | Exon | Synonymous | NM_152618.2:c.1380G>C | p.= | rs13135766 | 0.11 | Yes | Yes | Yes | Yes |
| #2 | *BBS12* | Subs. | Exon | Missense | NM_152618.2:c.1399G>A | p.Asp467Asn | rs13135778 | 0.11 | Yes | Yes | Yes | Yes |
| #2 | *BBS12* | Subs. | Exon | Synonymous | NM_152618.2:c.1410C>T | p.= | rs13135445 | 0.14 | Yes | Yes | Yes | Yes |
| #2 | *BBS12* | Subs. | Exon | Synonymous | NM_152618.2:c.1872A>G | p.= | rs13102440 | 0.11 | Yes | Yes | Yes | Yes |
| #2 | *BBS2* | Subs. | Exon | Synonymous | NM_031885.3:c.209G>A | p.= | rs4784677 | 0.004 | Yes | Yes | No | Yes |
| #2 | *BBS4* | Subs. | Intron | No | NM_033028.4:c.77-6G>A | p.? | rs8033604 | 0.05 | Yes | Yes | Yes | Yes |
| #2 | *BBS4* | Subs. | Exon | Synonymous | NM_033028.4:c.906T>C | p.= | rs12914333 | 0.03 | Yes | Yes | Yes | Yes |
| #2 | *BBS4* | Subs. | Exon | Missense | NM_033028.4:c.1061T>C | p.Ile354Thr | rs2277598 | 0.45 | Yes | Yes | Yes | Yes |
| #2 | *BBS9* | Subs. | Exon | Synonymous | NM_198428.2:c.999A>G | p.= | NA | NA | No | No | Yes | No |
| #2 | *BDNF* | Subs. | Exon | Synonymous | NM_170735.5:c.450A>G | p.= | rs2353512 | 0.005 | Yes | Yes | Yes | Yes |
| #2 | *CEL* | Subs. | Exon | Synonymous | NM_001807.4:c.1719C>T | p.= | rs488087 | 0.24 | Yes | No | No | Yes |
| #2 | *CEP290* | Indel | Intron | No | NM_025114.3:c.3574-9del | p.? | rs10717563 | NA | Yes | Yes | No | Yes |
| #2 | *CEP290* | Indel | Intron | No | NM_025114.3:c.2484-8_2484-4delinsGTTTT | p.? | NA | NA | Yes | Yes | Yes | Yes |
| #2 | *CEP290* | Subs. | Exon | Synonymous | NM_025114.3:c.4119A>G | p.= | rs117122459 | 0.02 | Yes | Yes | No | Yes |
| #2 | *CEP290* | Subs. | Exon | Missense | NM_025114.3:c.2512A>G | p.Lys838Glu | rs11104738 | 0.11 | Yes | Yes | Yes | Yes |
| #2 | *CEP290* | Subs. | Exon | Synonymous | NM_025114.3:c.2268A>G | p.= | rs2468255 | 0.2 | Yes | Yes | No | Yes |
| #2 | *EIF2AK3* | Indel | Exon | Missense | NM_004836.5:c.61_63delins--- | p.Leu21 | NA | NA | Yes | No | Yes | Yes |
| #2 | *EIF2AK3* | Subs. | Exon | Missense | NM_004836.5:c.2110G>T | p.Ala704Ser | rs1805165 | 0.29 | Yes | Yes | Yes | Yes |
| #2 | *EIF2AK3* | Subs. | Exon | Synonymous | NM_004836.5:c.1791A>G | p.= | rs1805164 | 0.29 | Yes | Yes | Yes | Yes |
| #2 | *EIF2AK3* | Subs. | Intron | No | NM_004836.5:c.1763+6A>T | p.? | rs6750998 | 0.2 | Yes | Yes | Yes | Yes |
| #2 | *EIF2AK3* | Subs. | Exon | Missense | NM_004836.5:c.497A>G | p.Gln166Arg | rs13045 | 0.35 | Yes | Yes | Yes | Yes |
| #2 | *GLIS3* | Subs. | Exon | Missense | NM_152629.3:c.902C>A | p.Pro301Gln | rs6415788 | 0.31 | Yes | No | Yes | Yes |
| #2 | *GLIS3* | Subs. | Exon | Missense | NM_152629.3:c.805T>C | p.Ser269Pro | rs806052 | 0.002 | Yes | No | Yes | Yes |
| #2 | *GNAS* | Subs. | Exon | Synonymous | NM_000516.4:c.393C>T | p.= | rs7121 | 0.39 | Yes | Yes | Yes | Yes |
| #2 | *HNF1A* | Subs. | Exon | Synonymous | NM_000545.5:c.51C>G | p.= | rs1169289 | 0.41 | Yes | Yes | Yes | Yes |
| #2 | *HNF1A* | Subs. | Exon | Synonymous | NM_000545.5:c.864G>C | p.= | rs56348580 | 0.21 | Yes | Yes | Yes | Yes |
| #2 | *HNF1A* | Subs. | Exon | Synonymous | NM_000545.5:c.1720A>G | p.= | rs1169305 | 0.01 | Yes | Yes | Yes | Yes |
| #2 | *HNF1B* | Indel | Exon | In-frame | NM_000458.2:c.207_211delins----- | p.His69_Lys71delinsArg | NA | NA | Yes | Yes | Yes | Yes |
| #2 | *INS* | Subs. | 3'UTR | No | NM_000207.2:c.*9C>T | p.? | rs3842752 | 0.14 | Yes | Yes | No | Yes |
| #2 | *LEPR* | Subs. | Exon | Missense | NM_002303.5:c.326A>G | p.Lys109Arg | rs1137100 | 0.39 | Yes | No | No | Yes |
| #2 | *LEPR* | Subs. | Exon | Missense | NM_002303.5:c.668A>G | p.Gln223Arg | rs1137101 | 0.41 | Yes | Yes | No | Yes |
| #2 | *LEPR* | Subs. | Exon | Synonymous | NM_002303.5:c.3057G>A | p.= | rs1805096 | 0.46 | Yes | Yes | Yes | Yes |
| #2 | *MKS1* | Subs. | Intron | No | NM_017777.3:c.858+9A>G | p.? | rs3826300 | 0.45 | Yes | Yes | Yes | Yes |
| #2 | *NEUROD1* | Subs. | Exon | Missense | NM_002500.4:c.590C>A | p.Pro197His | rs8192556 | 0.01 | Yes | Yes | Yes | Yes |
| #2 | *NEUROD1* | Subs. | Exon | Synonymous | NM_002500.4:c.133A>G | p.= | rs1801262 | 0.27 | Yes | Yes | Yes | Yes |
| #2 | *NEUROG3* | Subs. | Exon | Missense | NM_020999.3:c.596T>C | p.Phe199Ser | rs4536103 | 0.45 | Yes | Yes | Yes | Yes |
| #2 | *PCSK1* | Subs. | Exon | Synonymous | NM_000439.4:c.1650T>C | p.= | rs6233 | 0.34 | Yes | Yes | No | Yes |
| #2 | *PDX1* | Subs. | Exon | Missense | NM_000209.3:c.788C>A | p.Pro263His | NA | NA | No | Yes | No | No |
| #2 | *PTF1A* | Subs. | Exon | Missense | NM_178161.2:c.787T>C | p.Ser263Pro | rs7918487 | 0.38 | Yes | No | Yes | Yes |
| #2 | *PTF1A* | Subs. | Exon | Missense | NM_178161.2:c.203T>C | p.Leu68Pro | NA | NA | No | Yes | No | No |
| #2 | *SDCCAG8* | Subs. | Exon | Missense | NM_006642.3:c.1134A>T | p.Glu378Asp | NA | NA | Yes | Yes | Yes | Yes |
| #2 | *WDPCP* | Subs. | Intron | No | NM_015910.5:c.1916-6C>T | p.? | rs2421862 | 0.12 | Yes | Yes | Yes | Yes |
| #2 | *WFS1* | Subs. | Intron | No | NM_006005.3:c.461-9A>G | p.? | rs10010131 | 0.27 | Yes | Yes | Yes | Yes |
| #2 | *WFS1* | Subs. | Exon | Synonymous | NM_006005.3:c.684C>G | p.= | rs1801213 | 0.24 | Yes | Yes | Yes | Yes |
| #2 | *WFS1* | Subs. | Exon | Missense | NM_006005.3:c.997G>A | p.Val333Ile | rs1801212 | 0.14 | Yes | Yes | Yes | Yes |
| #2 | *WFS1* | Subs. | Exon | Synonymous | NM_006005.3:c.1185C>T | p.= | rs1801206 | 0.33 | Yes | Yes | Yes | Yes |
| #2 | *WFS1* | Subs. | Exon | Synonymous | NM_006005.3:c.1500C>T | p.= | NA | NA | Yes | Yes | Yes | Yes |
| #2 | *WFS1* | Subs. | Exon | Missense | NM_006005.3:c.1591C>G | p.Leu531Val | NA | NA | Yes | Yes | Yes | Yes |
| #2 | *WFS1* | Subs. | Exon | Missense | NM_006005.3:c.1832G>A | p.Arg611His | rs734312 | 0.48 | Yes | Yes | Yes | Yes |
| #2 | *WFS1* | Subs. | Exon | Synonymous | NM_006005.3:c.1833C>G | p.= | NA | NA | Yes | Yes | Yes | Yes |
| #2 | *WFS1* | Subs. | Exon | Synonymous | NM_006005.3:c.2433G>A | p.= | rs1046314 | 0.32 | Yes | Yes | Yes | Yes |
| #2 | *WFS1* | Subs. | Exon | Synonymous | NM_006005.3:c.2565A>G | p.= | NA | NA | Yes | Yes | No | Yes |

***MAF***, minor allele frequency (global MAF according to dbSNP database)**; *Subs.***, substitution.
